# Exploring Runs of Homozygosity and Heterozygosity in Sheep Breeds Maintained in Poland

**DOI:** 10.3390/genes16060709

**Published:** 2025-06-14

**Authors:** Tomasz Szmatola, Katarzyna Ropka-Molik, Igor Jasielczuk, Aldona Kawęcka, Artur Gurgul

**Affiliations:** 1 Department of Basic Sciences, University of Agriculture in Krakow, Rędzina 1c, 30-248 Kraków, Poland; igor.jasielczuk@urk.edu.pl (I.J.); artur.gurgul@urk.edu.pl (A.G.); 2 Department of Animal Molecular Biology, National Research Institute of Animal Production, Krakowska 1, 32-083 Balice, Poland; katarzyna.ropka@iz.edu.pl; 3 Department of Sheep and Goat Breeding, National Research Institute of Animal Production, Krakowska 1, 32-083 Balice, Poland; aldona.kawecka@iz.edu.pl

**Keywords:** inbreeding coefficient, autozygosity, ROH, breed diversity

## Abstract

**Objectives**: The study investigates runs of homozygosity (ROH) and heterozygosity (ROHet), and their patterns in nine sheep breeds (772 animals in total) maintained in Poland (native and conserved), corresponding to their genetic diversity, inbreeding levels, and selection signatures. **Methods**: Genotypes were obtained using the Illumina OvineSNP50 BeadChip and quality-filtered SNPs were used to detect ROH and ROHet segments with the detectRUNS R package, following stringent parameters for segment length, SNP density, and genotype quality. **Results**: Significant variation in ROH characteristics was observed across breeds. Short ROH segments were predominant in all breeds, indicating historical inbreeding events. In contrast, longer ROH segments signified recent inbreeding, particularly in Swiniarka (SW) and Polish Merino of Colored Variety (MPC). The ROH-based genomic inbreeding coefficient (F_ROH_) varied across breeds, with SW exhibiting the highest levels, suggesting reduced genetic diversity. ROHet analysis revealed that Uhruska (UHR) had the highest heterozygous segments span, while Black-headed (BH) sheep exhibited the lowest ROHet extent. ROH islands identified across breeds revealed regions under selection, associated with traits such as reproductive performance, wool quality, and body condition. Genes located within these islands (e.g., *U6*, *SPP1*, *ABCG2*) were linked to economically significant traits including milk production, growth, and carcass quality. **Conclusions**: The presented results highlight the genetic adaptations shaped by selection pressures, while also providing insights into the genetic architecture of sheep breeds maintained in Poland.

## 1. Introduction

Runs of homozygosity (ROH) are contiguous genomic segments where both alleles are identical, typically arising due to inbreeding. These regions are valuable indicators of genetic diversity, population structure, and the selection pressure observed within studied populations [1]. The identification and analysis of ROH provide critical insights into the genetic status of populations, including the potential risks of inbreeding depression and the accumulation of deleterious alleles [2,3]. The study of ROH has become a valuable approach for understanding the genetic structure of livestock species. In sheep, ROH analysis has proven essential not only in assessing the impact of selective breeding on genetic diversity, but also in identifying genomic regions under selection and evaluating population structure, demographic history, and inbreeding levels. For example, Kizilaslan [4] demonstrated that prolonged inbreeding leads to an increase in autozygosity, reflected in the formation of long ROH segments within the genome. This phenomenon is particularly evident in breeds subjected to intense selection pressures, where the focus on specific traits often reduces overall genetic variability. Such reductions in genetic diversity can have significant consequences, influencing not only productivity but also the ability of sheep to adapt to changing environmental conditions [4].

ROH can form regions known as ROH islands, which are segments of the genome with a high frequency of occurrences within a specific population. These regions are primarily shaped by strong selection pressures and frequently overlap with regions under positive selection [5]. ROH islands often harbor genes associated with traits of evolutionary, economic, or adaptive significance, such as disease resistance, reproductive traits, or environmental adaptation [5]. Identifying these islands provides valuable insights into the genetic structure of a population and highlights loci influenced by selective breeding or environmental factors [6,7]. Due to this, associations between ROH characteristics and phenotypic traits have recently become a major topic in livestock genetics research [5,6,8,9]. Dlamini et al. [10] identified ROH patterns in sheep populations selected for parasite resistance, revealing that regions of high homozygosity overlapped with quantitative trait loci (QTL) linked to resistance traits. These findings highlight the utility of ROH analyses in identifying genomic regions under selection, which can be targeted in breeding programs to enhance desirable traits.

The genetic diversity of sheep breeds is also shaped by historical factors such as domestication time and geographical distribution. Abied et al. [11] utilized ROH analyses to investigate the demographic history and population structure of Chinese sheep breeds, providing valuable insights into how these factors have influenced their genetic landscape. Understanding the historical context of genetic variation is essential for developing effective conservation and breeding strategies, particularly in response to modern challenges such as climate change and disease outbreaks. Beyond its applications in breeding and conservation, ROH analysis holds broader implications for the livestock industry. Identifying selection signatures within ROH regions can guide management practices aimed at balancing genetic diversity with the optimization of production traits. For instance, Ghoreishifar et al. [12] highlighted how ROH patterns could inform breeding decisions to mitigate inbreeding risks and enhance the resilience of sheep populations.

In addition to ROH, there has been growing interest in investigating runs of heterozygosity (ROHet), defined as extended genomic regions with high heterozygosity across consecutive SNPs. While their biological interpretation remains under discussion—particularly regarding whether they constitute true ‘runs’—recent studies have operationalized ROHet to explore heterozygosity-rich regions associated with outbreeding, balancing selection, or gene flow. This approach has provided valuable insights into genetic diversity and adaptive variation in livestock populations [13,14]. In our study, we include ROHet analysis to complement the assessment of inbreeding and selection in sheep breeds maintained under conservation and production systems. In this study, we aim to investigate the distribution and characteristics of runs of homozygosity and heterozygosity across nine sheep breeds maintained in Poland, assess genomic inbreeding levels, and identify regions of the genome under selection pressure through the analysis of ROH islands. This multifaceted approach contributes to understanding breed diversity, historical inbreeding, and potential selection signatures relevant to economically important traits. In addition, ROH islands were identified and compared with a QTL annotation database to reveal potential selection signatures and their functional implications. Moreover, the F_ROH_ (genomic inbreeding coefficient) is estimated, allowing for the assessment of inbreeding levels in selected sheep populations, of which some are under conservation breeding programs in which maximization of genetic diversity is crucial.

## 2. Material and Methods

### 2.1. Study Material, DNA Isolation and Genotyping, and Filtering of Genotypic Data

The study utilized DNA extracted from blood samples of 772 sheep (primarily females) representing nine distinct breeds or varieties: Polish Mountain Sheep (PMS, *n* = 104), Podhale Zackel (PZ, *n* = 100), Colored Mountain Sheep (CMS, *n* = 97), Świniarka (SW, *n* = 99), Uhruska (UHR, *n* = 69), Old-type Merino (MPOT, *n* = 50), Polish Merino of Colored Variety (MPC, *n* = 59), Black-headed (BH, *n* = 104), and Wrzosówka (WRZ, *n* = 90). All these breeds are a part of the national conservation program, with their general characteristics summarized in Table 1 (based on [15]). This dataset was partially utilized in our previous report on sheep selection signatures, in which a comprehensive description of the origin, recent conservation status, and physical appearance of each breed is provided [15]. Additional insights into genetic differentiation, historical effective population size, and haplotype blocks are available in Jasielczuk et al. [16].

Blood samples were obtained from at least two separate farms per breed participating in the conservation programs. All procedures involving animals were conducted in accordance with European Union regulations and received approval from the Local Animal Care Ethics Committee No. II in Krakow (Permit number 1293/2016). DNA purification was performed using the QuickGene DNA whole blood kit S (DB-S; Kurabo, Osaka, Japan), and the quality of the extracted DNA was evaluated using a Nanodrop2000 spectrophotometer (Thermo Fisher Scientific, Waltham, MA, USA). The purified DNA was then diluted to the required concentration of 50 ng/µL, with 200 ng utilized for genotype analysis.

DNA samples were processed using Infinium Ultra arrays with the OvineSNP50 DNA Analysis Kit (Illumina, San Diego, CA, USA), facilitating the genotyping of 54,241 SNPs. The genotyping procedure included DNA amplification, enzymatic fragmentation, purification, hybridization to the arrays, probe extension, and staining. The processed arrays were then scanned using the HiScanSQ System (HiScanSQ System 2000) (Illumina). Genotypes were extracted using GenomeStudio software (version 2.0) (Illumina), and only samples with call rates exceeding 0.95 were included in the analysis. The SNP positions were subsequently remapped to the ISCG Oar_v3.1 genome assembly using the UCSC LiftOver tool. Although more recent sheep genome assemblies are available (e.g., Oar_rambouillet_v1.0, ARS-UI_Ramb_v2.0), we used Oar_v3.1 due to its widespread application in previous ovine genomic studies and its compatibility with reference annotations. This ensured the consistency and interpretability of QTL mapping and gene annotations across ROH regions. SNPs located on the Y chromosome, as well as unmapped SNPs, were excluded. The resulting set of 50,568 SNPs was further filtered for polymorphism and quality: 271 SNPs were removed due to missing genotype data (>20%), and 2106 SNPs were excluded due to a minor allele frequency below 5%. Additionally, SNPs were filtered for Hardy–Weinberg equilibrium by identifying and removing deviating SNPs (*p* < 1.0 × 10^−5^) in each population separately. This quality control process resulted in a final set of 48,090 common SNPs with an average genotyping rate of 99%, which was used for downstream analysis.

### 2.2. Identification of Runs of Homozygosity and Heterozygosity, and Estimation of Genomic Inbreeding

The identification of runs of homozygosity (ROH) and heterozygosity (ROHet), along with the estimation of genomic inbreeding coefficients, was performed using the detectRUNS R package (v.0.9.6), employing the consecutive SNP-based run detection method as outlined by Biscarini et al. [17]. ROH detection parameters were selected following recommendations by Biscarini et al. [17] and previous livestock studies using medium-density arrays. Parameters were tuned to ensure compatibility with the SNP density of the OvineSNP50 array and to maintain reliability in identifying both short and long ROH segments. ROH detection criteria included a minimum of 30 consecutive homozygous SNPs, a minimum ROH length of 0.5 Mb, a maximum SNP spacing of 1 Mb, and allowances of up to 1 SNP with a heterozygous genotype and 1 SNP with a missing genotype. For ROHet detection, where no standardized criteria are available yet, guidelines from [17,18,19] were applied. Specifically, the criteria included a minimum of 15 consecutive homozygous SNPs, a minimum ROHet length of 1 Mb, a maximum SNP distance of 1 Mb, with up to 3 SNPs with homozygous genotypes and 2 SNPs with missing genotypes permitted.

ROH were categorized into five length-based classifications: >1 Mb, >2 Mb, >4 Mb, >8 Mb, and >16 Mb, while ROHet classifications were set at >0.5 Mb, >1 Mb, and >1.5 Mb. For both ROH and ROHet, the average sums per length category were calculated by summing all ROH or ROHet identified per animal within each category and averaging the values. Genomic inbreeding coefficients (F_ROH_) were estimated as in the McQuillan et al. [20] by dividing the total ROH length within each selected category by the sum of autosomal chromosome lengths covered by SNPs. The ROH length categories employed in this calculation were >1 Mb, >2 Mb, >4 Mb, >8 Mb, and >16 Mb [1]. For ROHet, we calculated D_ROHet_ parameters for >0.5 Mb, >1 Mb, and >1.5 Mb following Biscarini et al. [17].

To assess the statistical differences between studied sheep breeds in respect to the number and sum of ROH lengths, the Wilcoxon rank-sum test was used. The Wilcoxon rank-sum test was chosen due to the non-normal distribution of ROH count and length data, as determined by the Shapiro–Wilk normality test. This non-parametric approach allowed for robust comparison between breeds without assuming data normality.

#### Identification of ROH Hotspot Regions and Association with QTL Database

To identify hotspot regions of runs of homozygosity (ROH), we initially calculated the frequency with which each SNP appeared within ROH across the dataset. Then, the top 1% of SNPs with the highest occurrence in ROH were selected and neighboring SNPs were aggregated into continuous regions, referred to as ROH hotspots. All identified ROH hotspots were subsequently analyzed for overlapping genes using Ensembl BioMart (https://www.ensembl.org/biomart/martview/43e616ef833bc13d998d33fc3804e4ce, (accessed on 2 February 2025) based on Ensembl gene version 111. Additionally, to evaluate the biological processes and molecular functions associated with these genes, Kobas-I software (version 3.0) was employed [21].

ROH hotspots were matched against trait-linked QTL obtained from the Animal QTL Database (https://www.animalgenome.org/cgi-bin/QTLdb/index, accessed on 2 February 2025), and overlapping QTL regions were identified. This approach allowed us to examine whether ROH islands coincided with known QTL relevant to economically important traits.

## 3. Results

### 3.1. Number of ROH per Animal

The analysis of the distribution of runs of homozygosity within nine studied sheep breeds revealed substantial variation in different ROH characteristics that are presented below.

The mean number of ROH segments per animal differed significantly across most breeds and ROH length categories. For the >1 Mb category, MPC and MPOT had the highest mean ROH counts per animal (86.98 ± 9.95 and 57.68 ± 8.52, respectively), while PZ showed the lowest mean ROH count (13.11 ± 5.21).

**Figure 1:**
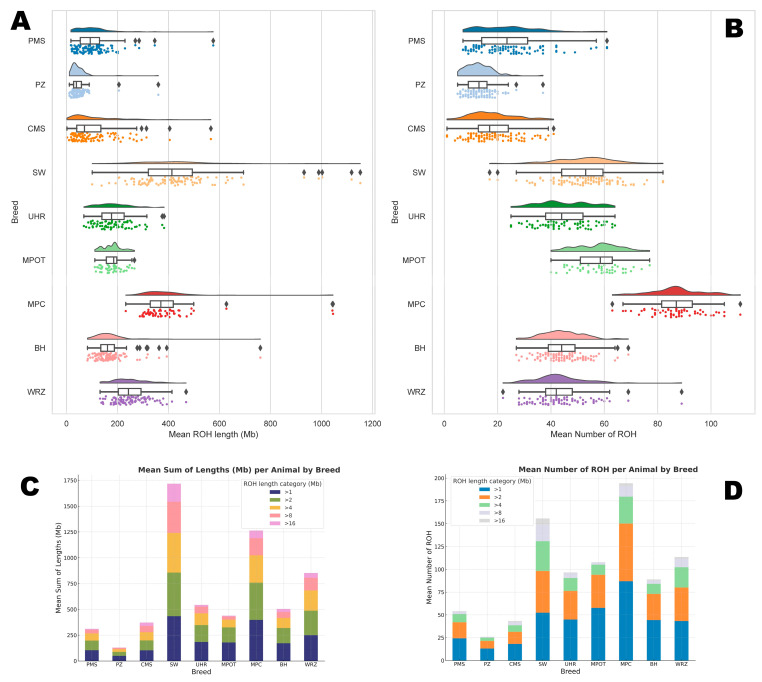
The number and length of runs of homozygosity per animal for each sheep breed. (**A**) represents the sum of all ROH lengths per animal; (**B**) represents the amount of all ROH per animal; (**C**) is the distribution of ROH lengths in respect to ROH length categories; (**D**) is the distribution of ROH numbers in respect to ROH length category. PMS—Polish Mountain Sheep; PZ—Podhale Zackel; CMS—Colored Mountain Sheep; SW—Swiniarka; UHR—Uhruska; MPOT—Old-type Merino; MPC—Polish Merino of Colored Variety; BH—Black-headed; WRZ—Wrzosówka.

With increasing ROH lengths, all breeds showed a general decline in mean ROH counts per animal, indicating that in all studied breeds, shorter ROH segments are more prevalent. Notably, SW exhibited the highest mean count of long ROH segments (>16 Mb) at 6.88 ± 4.93, while breeds such as PZ and MPOT had very low counts in this category (0.13 ± 0.66 and 0.36 ± 0.63, respectively), suggesting limited recent inbreeding in the latter breeds. The basic statistics for these observations are graphically presented in Figure 1, and in detail in Supplementary Material S1 (Table S1).

### 3.2. Sums of ROH Lengths per Animal

The total ROH length per animal also varied across breeds. SW displayed the highest mean sum of ROH lengths per animal in the >1 Mb category (433.42 Mb ± 184.32), while MPC followed closely with a mean ROH length of 398.23 Mb ± 137.88. In contrast, PZ had the shortest ROH length in this category (48.82 Mb ± 40.20).

With each subsequent ROH length category, total ROH lengths decreased across breeds. In the long >16 Mb ROH category, SW showed the highest total ROH length per animal (175.63 Mb ± 153.18), indicative of likely more recent inbreeding. In contrast, UHR and MPOT showed lower total ROH lengths (16.75 Mb ± 24.26 and 8.13 Mb ± 15.83, respectively). The basic statistics for these observations are graphically presented in Figure 1, and in detail in Supplementary Material S1 (Table S1).

The Wilcoxon test results regarding the comparisons between breeds in the sums of ROH lengths and ROH numbers are presented in Supplementary Material S2.

### 3.3. Assessment of Genomic Inbreeding Levels

**Figure 2:**
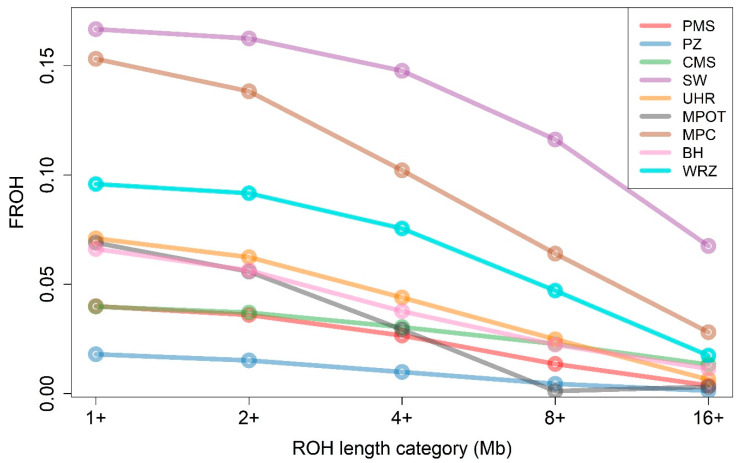
The proportion of the genome covered by runs of homozygosity (F_ROH_) across different ROH length categories and sheep breeds. PMS—Polish Mountain Sheep; PZ—Podhale Zackel; CMS—Colored Mountain Sheep; SW—Swiniarka; UHR—Uhruska; MPOT—Old-type Merino; MPC—Polish Merino of Colored Variety; BH—Black-headed; WRZ—Wrzosówka.

The analysis of genomic inbreeding based on the fraction of the genome in runs of homozygosity (F_ROH_) revealed varying levels of homozygosity among the different sheep breeds and ROH length categories (1+, 2+, 4+, 8+, and 16+ Mb). Notably, the SW breed exhibited the highest F_ROH_ values across all length categories, ranging from 0.1667 in the >1 Mb category to 0.0676 in the >16 Mb category. MPC also showed high levels of F_ROH_, with values between 0.1532 and 0.0281 across the different length categories. Conversely, the PZ breed displayed the lowest F_ROH_, with values starting at 0.0180 for ROH > 1 Mb and decreasing to 0.0013 in ROH > 16 Mb. Other breeds, including PMS, UHR, and MPOT, exhibited moderate F_ROH_ values across categories, indicating varying levels of homozygosity that were generally lower than those observed in SW and MPC but higher than those in PZ. The mean values of F_ROH_ for all nine breeds are graphically presented in Figure 2 and in Supplementary Material S1 (Table S2).

To further explore the heterozygosity landscape across the studied sheep breeds, the proportion of the autosomal genome covered by runs of heterozygosity (D_ROHet_) was calculated for three segment length thresholds: >0.5 Mb, >1 Mb, and >1.5 Mb. These values represent the fraction of the genome occupied by ROHet, analogous to F_ROH_ values for homozygous segments. Among all breeds, UHR exhibited the highest D_ROHet_ values across all three length categories, with 0.003908 for >0.5 Mb, 0.003590 for >1 Mb, and 0.001677 for >1.5 Mb. PMS also showed relatively elevated D_ROHet_ values (0.003235 in >0.5 Mb), followed closely by BH and CMS. However, for longer ROHet segments (>1.5 Mb), only UHR and PMS maintained notably high values, suggesting that long heterozygous regions are less common in most breeds. In contrast, breeds such as MPOT and MPC demonstrated consistently low D_ROHet_ values across all categories, particularly in the >1.5 Mb range (0.000348 and 0.000429, respectively). The mean values of D_ROHet_ for all nine breeds are graphically presented in Supplementary Material S1 (Table S2).

## 4. Distribution of ROHet

### 4.1. ROHet Segment Counts per Animal

**Figure 3:**
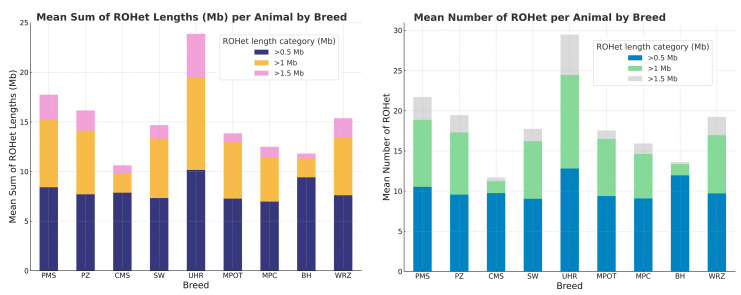
The number and length of runs of heterozygosity (ROHet) per animal for each sheep breed in respect to ROHet length category. PMS—Polish Mountain Sheep; PZ—Podhale Zackel; CMS—Colored Mountain Sheep; SW—Swiniarka; UHR—Uhruska; MPOT—Old-type Merino; MPC—Polish Merino of Colored Variety; BH—Black-headed; WRZ—Wrzosówka.

Across breeds, the mean number of ROHet segments per animal generally decreased with increasing segment length categories. For segments longer than 0.5 Mb, the mean number of ROHet segments per animal varied from 9.08 (MPC) to 12.81 (UHR). In the category exceeding 1 Mb, mean counts ranged from a minimum of 1.4 (BH) to a maximum of 11.65 (UHR). In the longest category (>1.5 Mb), the counts were generally the lowest, ranging from 0.25 (BH) to 5.0 (UHR). The UHR breed consistently exhibited the highest average number of ROHet segments across all length categories. Conversely, the BH breed had the lowest average counts in the categories above 1 Mb and 1.5 Mb. The basic statistics for these observations are presented in detail in Supplementary Material S1 (Table S3) and graphically in Figure 3.

### 4.2. ROHet Segment Lengths per Animal

The mean total length of ROHet segments per animal declined as the segment length category increased. Specifically, for segments longer than 0.5 Mb, the mean total ROHet length ranged between 6.96 Mb (MPC) and 10.16 Mb (UHR). In the >1 Mb category, mean segment lengths ranged from 1.4 Mb (BH) to 9.33 Mb (UHR). For segments exceeding 1.5 Mb, total segment lengths per animal were generally lowest, varying between 0.25 Mb (BH breed) and 4.36 Mb (UHR). Moreover, UHR breed consistently showed the highest mean total ROHet lengths across all segment categories, peaking at 19.44 Mb in the >0.5 Mb category. In contrast, BH sheep demonstrated the lowest mean ROHet lengths, particularly in the >1 Mb and >1.5 Mb categories.

Notable differences among breeds were also evident in maximum ROHet length sums. Breeds such as PMS, PZ, and WRZ exhibited high maximum ROHet lengths (up to approximately 17 Mb) within the >0.5 Mb category, whereas the BH breed displayed consistently low maximum values across all evaluated categories. The basic statistics for these observations are presented in detail in Supplementary Material S1 (Table S3) and graphically in Figure 3.

## 5. ROH Islands

### ROH Island Distribution

**Figure 4:**
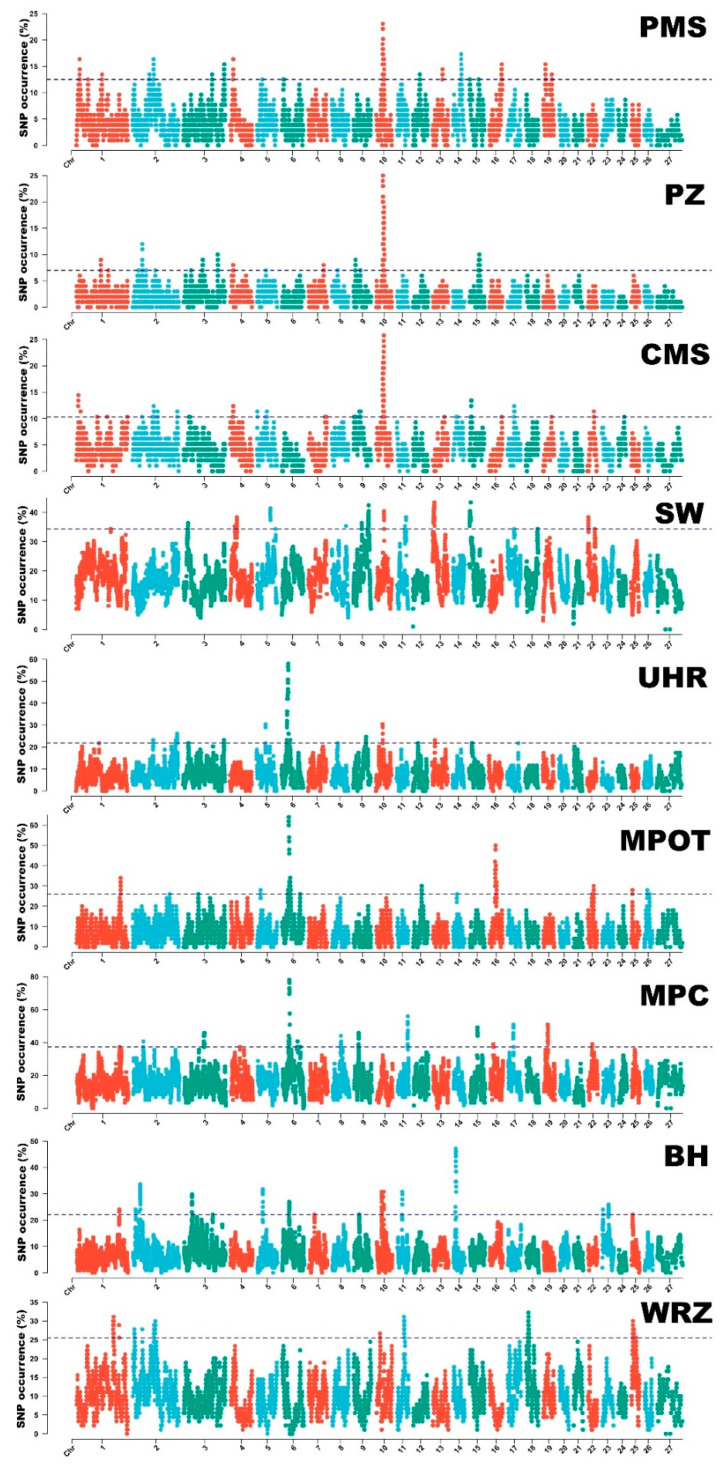
ROH patterns in all studied sheep breeds. The dashed line represents 1% of the highest ROH occurrences in a specific breed. PMS—Polish Mountain Sheep; PZ—Podhale Zackel; CMS—Colored Mountain Sheep; SW—Swiniarka; UHR—Uhruska; MPOT—Old-type Merino; MPC—Polish Merino of Colored Variety; BH—Black-headed; WRZ—Wrzosówka. ROH islands show gene and QTL contents.

Runs of homozygosity islands were identified for all nine studied sheep breeds. The SNPs most commonly occurring in ROH formed from 11 (UHR)–40 (CMS) continuous ROH islands (ROH hotspots) in the analyzed breed. The longest ROH hotspot region was identified in UHR, spanning over 184 SNPs and 9.46 Mb of genome sequence, followed by SW with 113 SNPs over 6.03 Mb, and MPOT with 107 SNPs across 5.5 Mb. Detailed information on these regions can be found in Supplementary Material S3. The distribution of ROH hotspots is graphically presented in Figure 4.

**Figure 5:**
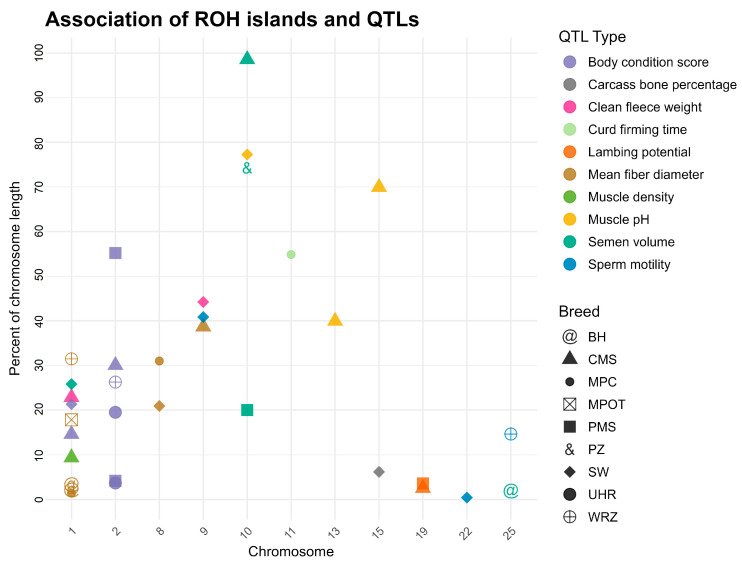
Co-localization of ROH islands with known QTL regions for studied sheep breeds. Y axis represents the percent of a chromosome length. Only chromosomes on which overlaps between ROH islands and QTL were identified are presented on the graph. PMS—Polish Mountain Sheep; PZ—Podhale Zackel; CMS—Colored Mountain Sheep; SW—Swiniarka; UHR—Uhruska; MPOT—Old-type Merino; MPC—Polish Merino of Colored Variety; BH—Black-headed; WRZ—Wrzosówka.

The identified ROH islands overlapped with several quantitative trait loci (QTL) that correspond to important phenotypic traits, including reproductive performance, wool characteristics, and body condition scores. Notably, the ROH hotspots in the CMS breed overlapped with the greatest number of QTL regions, including large QTL linked to semen volume on chromosome 10 (approximately 4.9 Mb) and muscle pH on chromosomes 13 and 15 (around 2.0 Mb and 3.5 Mb, respectively). Similarly, in the PZ breed, ROH islands spanned over a vast QTL region associated with semen volume (~3.7 Mb) also located on chromosome 10. Body condition score QTL were frequently detected in ROH hotspots across breeds, prominently on chromosome 2, involving breeds PMS, CMS, SW, UHR, and WRZ, suggesting its broad relevance in multiple populations. QTL regions influencing wool quality, such as mean fiber diameter, appeared consistently on chromosomes 1, 8, and 9 in breeds MPC, MPOT, BH, and WRZ, reflecting trait-specific genomic hotspots. This information is shown in detail in Supplementary Material S4 and visually in Figure 5.

In PMS, several ROH islands were detected on chromosomes 2, 10, and 19, with sizes ranging from approximately 1.8 Mb to 2.7 Mb. Notably, the ROH islands identified within QTL associated with body condition score and lambing potential were noted, indicating a strong selection for these traits within this breed. Similarly, PZ exhibited a large ROH island on chromosome 10, spanning 3 to 7 Mb, overlapping with QTL for semen volume, further emphasizing the importance of reproductive traits in breeding programs. CMS demonstrated a diverse array of ROH islands across multiple chromosomes, that overlapped with QTL associated with a clean fleece weight and muscle density QTL. In the SW population, ROH islands were identified on chromosomes 1, 10, and 15, characterized with substantial sizes (up to 6.03 Mb) indicating long stretches of homozygosity that overlapped with a number of QTL associated with body condition score and muscle pH. The UHR and MPOT breeds also exhibited large ROH islands, particularly on chromosome 2, where QTL for body condition score were identified. Overall, the distribution of ROH islands across breeds and their overlaps with specific QTL underscores the complex interplay between genetic diversity, selection pressures, and trait expression in sheep.

**Figure 6:**
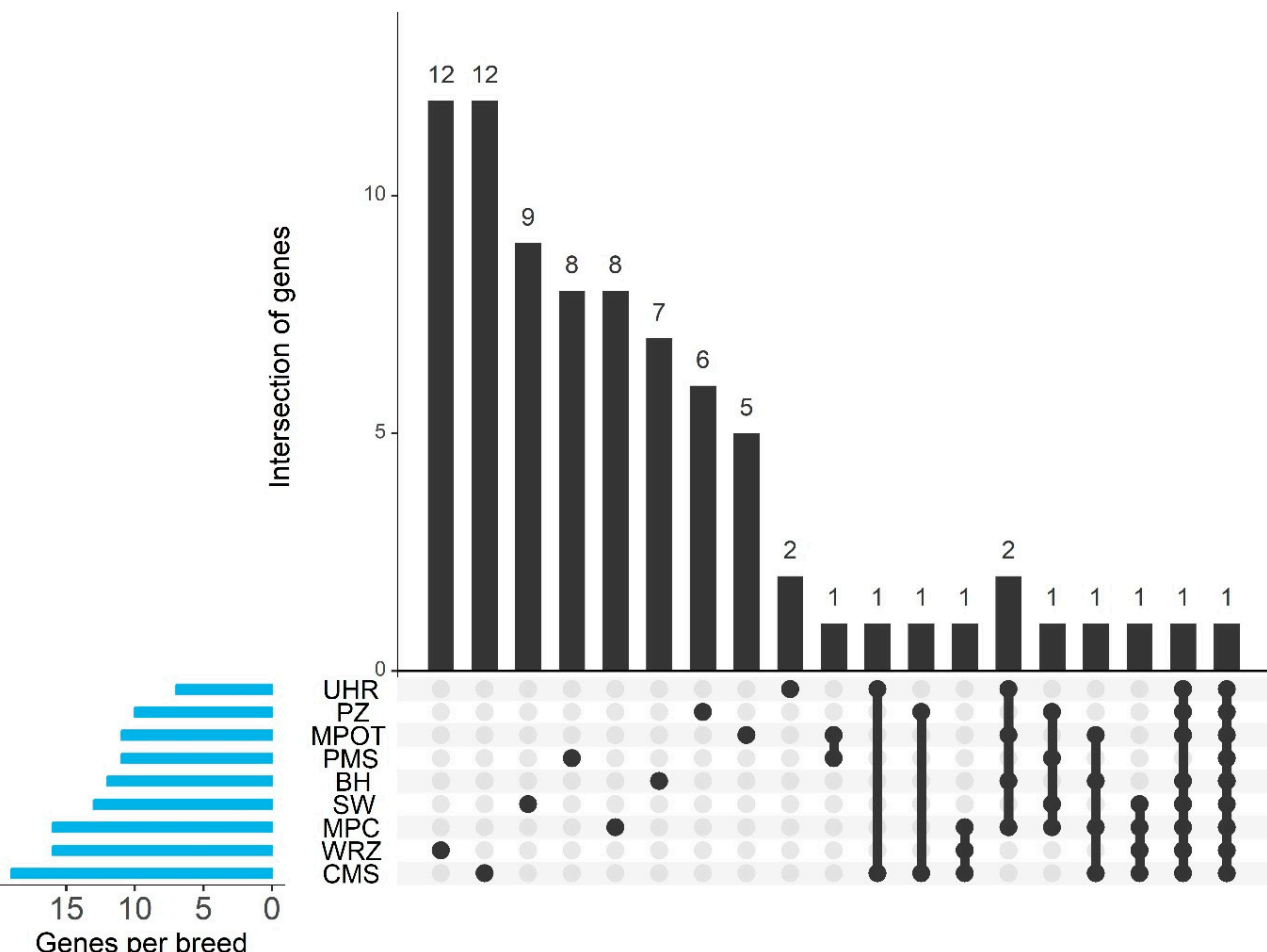
Gene intersection and breed-specific distribution of candidate genes identified in ROH islands. PMS—Polish Mountain Sheep; PZ—Podhale Zackel; CMS—Colored Mountain Sheep; SW—Swiniarka; UHR—Uhruska; MPOT—Old-type Merino; MPC—Polish Merino of Colored Variety; BH—Black-headed; WRZ—Wrzosówka.

Regions with high ROH occurrence overlapped with numerous identified genes, ranging from 7 in UHR to 19 in CMS. Additionally, most breeds had uniquely overlapped genes within their ROH islands, though 1–2 genes were common between breeds (Figure 6).

Several ROH islands were found to be shared among specific breeds, suggesting regions of potential common selection or ancestry. For instance, a prominent ROH region on chromosome 10 was observed in the PMS, PZ, and CMS breeds and includes the gene *EEF1AKMT1*. Another substantial ROH island, located on chromosome 6, was common to the UHR, MPOT, and MPC breeds; however, this region did not show a direct association with any known gene.

## 6. Discussion

### 6.1. Genetic Diversity of Polish Sheep Breeds

The analysis of runs of homozygosity (ROH) across nine sheep breeds revealed distinct patterns of genetic variation and inbreeding that can be potentially linked to each breed’s intended use and breeding history. Breeds such as MPC and MPOT exhibited the highest number of short ROH segments (1–4 Mb), suggesting historic or distant inbreeding events. In contrast, PZ demonstrated significantly fewer ROH segments which suggests greater genetic diversity.

As ROH segment length increased, all breeds consistently exhibited fewer ROH, highlighting the predominance of shorter segments that likely originated from more distant common ancestors. However, SW stood out with the highest count and length of long ROH segments (>16 Mb), as well as the highest F_ROH_ values across all ROH categories, suggesting more recent inbreeding and reduced genetic diversity. This trend was also reflected in the total length of ROH segments per animal, where SW and MPC again demonstrated the highest values, while PZ had the lowest ROH lengths. Notably, maximum ROH lengths also revealed breed-specific variation, with SW showing the most extensive homozygous regions, likely reflecting the small population size from which the breed was reconstituted.

### 6.2. Patterns of Runs of Homozygosity Across Polish Sheep Breeds in a Global Context

Overall, the results obtained in this study on Polish sheep breeds concerning ROH characteristics align with findings from other sheep breeds worldwide. Our analysis indicates that short ROH segments (<4 Mb) are predominant, consistent with the findings of Mastrangelo et al. [22] on Italian and Balkan sheep breeds, potentially indicating common ancestry or historical inbreeding across these breeds. This is particularly notable in breeds such as the MPC and SW. Additionally, breeds like SW, MPC, and CMS exhibit a high frequency of long ROH segments (>16 Mb), suggesting recent inbreeding events. This pattern, commonly observed in geographically isolated or intensively managed breeds, aligns with results from Granero et al. [23] on Spanish Merino sheep. The SW breed shows the highest ROH counts and lengths across all categories, particularly in the longer segments, suggesting limited genetic diversity—a finding that is consistent with Signer-Hasler [24], who reported similar patterns in heritage sheep breeds under conservation. SW displaying high coverage of the genome in ROH parallels endangered or isolated breeds such as the Valais Blacknose, which similarly exhibit elevated ROH counts in longer segments potentially due to restricted gene flow [24]. For the MPC breed, high ROH counts and mean lengths in shorter segments were observed. This pattern, also seen in studies on Merino derivatives [25], can be attributed to historical selection pressures for wool production, leading to the accumulation of shorter ROHs from older selective inbreeding. Comparable trends were observed in Spanish and Australian Merino breeds, which, while displaying high mean ROH in shorter segments, retain longer ROH due to intensive recent breeding for fiber traits [26]. In contrast, the PMS and PZ breeds exhibit relatively low numbers of ROH counts across all categories, particularly in the longest segments (>16 Mb), which is a strong indicator of greater genetic diversity and fewer recent inbreeding events. These results align with studies on less-managed or widely outcrossed breeds, such as the Scottish Blackface [27]. The findings for PMS and PZ suggest that they maintain a more diverse gene pool, potentially due to historical crossbreeding or lower selection pressure for specific traits, mirroring the diversity seen in resilient mountain-adapted or open-grazing sheep breeds [26]. The Uhruska breed shows moderate ROH counts and lengths, especially in the longest category (>16 Mb), indicating an intermediate level of genetic diversity. This pattern aligns with findings in semi-managed or partially crossbred breeds that retain moderate genetic diversity, comparable to breeds like the Karakachan sheep, as reported by Mastrangelo et al. [22]. Lastly, the Wrzosówka breed exhibits a tendency for fewer but longer ROH segments, similar to other native or rare breeds with limited genetic contributors. This breed’s pattern of long homozygous segments likely reflects lineage conservation, resembling endangered or heritage breeds in Europe, such as the Racka sheep, which show high homozygosity within a restricted genomic subset due to intensive conservation breeding efforts [28].

In this study, clear differences were observed in the number and total length of ROHet segments among the evaluated breeds.

### 6.3. Runs of Heterozygosity Profiles

Across all ROHet length categories, the UHR breed consistently displayed the highest number and total length of ROHet segments. This may reflect historical gene flow or selective breeding strategies that favored the maintenance of genetic diversity. In contrast, the BH breed showed the lowest ROHet counts and total lengths in the >1 Mb and >1.5 Mb categories, showing notably lower levels of high heterozygosity regions within the genome.

As expected, both the number and total length of ROHet segments decreased with increasing segment size, consistent with the notion that shorter heterozygous regions are more common and may represent older recombination events. Interestingly, maximum ROHet segment lengths varied considerably among breeds, with PMS, PZ, and WRZ demonstrating high maximum values in the >0.5 Mb category. These findings suggest that, while some breeds maintain extensive heterozygous segments likely due to more recent recombination, others may have experienced greater fixation due to inbreeding or selective pressure. Overall, the ROHet profiles observed within this study reflect the complex breeding histories and selection pressures that have shaped the genetic architecture of these traditional sheep breeds.

D_ROHet_ coefficients, which quantify the proportion of the genome covered by runs of heterozygosity, offer additional insights into the heterozygosity landscape across the studied sheep breeds. These values complement the FROH estimates by highlighting regions where heterozygosity has been maintained, potentially reflecting recombination, gene flow, or historical outcrossing. Notably, the UHR breed consistently exhibited the highest D_ROHet_ values across all segment length thresholds. This aligns with its highest ROHet counts and segment lengths, suggesting a genomic structure shaped by relatively low levels of recent inbreeding and potential past admixture. The preservation of long heterozygous segments in UHR may indicate a more diverse genetic base or gene flow from genetically distant sources—factors that can be advantageous in maintaining adaptability and reducing the risk of inbreeding depression. PMS and BH breeds also displayed elevated D_ROHet_ values, particularly in the shortest ROHet category (>0.5 Mb), indicating a broader spread of heterozygous regions across the genome. In contrast, MPOT and MPC showed the lowest D_ROHet_ levels, especially in the longest segment class (>1.5 Mb), which corresponds with their high ROH-based inbreeding (F_ROH_) values. These patterns support the interpretation that Merino-type breeds in this study have undergone more intensive selection and possible inbreeding, leading to greater genomic homozygosity and lower heterozygosity persistence.

Importantly, D_ROHet_ analysis serves as a valuable complement to ROH analysis in evaluating the genetic structure of populations. While ROH provide insight into inbreeding and autozygosity, ROHet-based metrics such as D_ROHet_ allow researchers to assess genomic regions where diversity has been retained. This dual approach offers a more nuanced understanding of the balance between inbreeding and genetic diversity, which is especially crucial for breeds under conservation programs.

### 6.4. ROH Islands

The results presented within this study provide insights into the ROH structure of sheep populations maintained in Poland. The presence, localization, and characteristics of ROH islands in specific breeds indicate a history of inbreeding and selection, which can have both positive and negative consequences for genetic diversity and overall population health. As noted by Ruiz-Larrañaga et al. [29], the persistence of selection signatures in the form of ROH hotspots can reveal historical selection events that have shaped the genetic landscape of sheep breeds. The identification of ROH associated with reproductive traits, such as lambing potential and semen volume, suggests that these traits have been prioritized in breeding programs, aligning with the economic goals of sheep production [30]. Moreover, the significant association of ROH islands with body condition scores across multiple breeds highlights the importance of this trait in sheep management [8,30]. The investigation of genes located in ROH islands in sheep offers potential insights into the genetic underpinnings of economically significant production traits, such as growth, meat quality, milk production, and wool characteristics. Genes identified across multiple sheep breeds (e.g., *U6*, *5S_rRNA*, *U2*, *ABCG2*, *SPP1*, *7SK*, *SNORA70*, *U4*, *ID3*, *RNase_MRP*, and *Metazoa_SRP*) have been associated with various production traits, as evidenced by the existing literature. For example, *U6* and *5S_rRNA* genes [27], integral components of the small nuclear RNA family, are critical in RNA processing and gene regulation. Studies by Rezvannejad et al. [31] and Li et al. [32] have highlighted these genes as selection signatures linked to milk production traits in sheep, suggesting their influence on lactation performance and milk composition. Their presence across multiple breeds indicates a potential universal role in milk production, mirroring findings in cattle where *5S_rRNA* has been positively selected for milk traits [8]. Notably, the ROH hotspot overlapping the *5S_rRNA* gene was detected in all breeds except PMS, underscoring its importance in milk production. Similarly, the *U2* gene, another small nuclear RNA family member, may contribute to regulatory functions that enhance milk yield and quality [33].

Some genes from the small nuclear RNA family (*U4*, *U12*, *U2*) were identified in the ROH patterns that contribute to milk production traits. These genes are known for their pivotal role across sheep, goats, and cattle [31]. The small nuclear RNA (snRNA) genes—U2, U4, and U12—play a crucial role in the splicing of pre-mRNA, a fundamental process for gene expression regulation. Their functional significance, as proper splicing is essential for the expression of key genes governing mammary gland development and lactation physiology. Studies in sheep, goats, and cattle have consistently highlighted their contribution to milk yield and composition, reinforcing their pivotal regulatory role in livestock productivity [34].

*ABCG2*, a gene involved in the transport of substances across cellular membranes, has also been linked to milk production traits in sheep. Gutiérrez-Gil et al. [35] identified *ABCG2* as a candidate gene for dairy production, implicating its role in the transport of milk components. This aligns with findings by Pazzola et al. [36], where *ABCG2* influenced milk fat content and overall dairy performance. Its identification in ROH islands of breeds such as BH, MPC, MPOT, and UHR underscores its significance in breeding programs aimed at improving milk traits.

Moreover, the *SPP1* gene, identified in ROH islands of BH, MPC, MPOT, known for its involvement in bone metabolism, carcass traits, and reproductive traits, has also been associated with milk production. Dettori et al. [37] noted that *SPP1* influences both milk yield and composition, indicating its dual role in lactation and reproductive success. The presence of *SPP1* in ROH hotspots of breeds selected for meat and wool-meat purposes suggests that selection pressures for these traits may have inadvertently favored its role in milk production as well. This dual selection could explain the higher prolificacy observed in certain breeds, as *SPP1* is also linked to reproductive efficiency [38]. The observed selection signatures on the *SPP1* gene in breeds selected for meat and wool-meat purposes indicate a complex interplay between selection for milk traits and other economically important traits such as growth and carcass quality. Furthermore, genes such as *7SK* (CMS, MPC, SW, WRZ) and *SNORA70* (MPC, PMS, PZ, SW), implicated in RNA processing, were identified in specific breeds and may contribute to the regulation of growth and metabolic processes. Their presence in ROH islands suggests selection for traits related to growth and feed efficiency, which are critical for meat production [39]. Similarly, genes like *U4*, *ID3*, *RNase_MRP*, and *Metazoa_SRP*, although less studied in sheep, likely play roles in cellular differentiation and metabolic pathways influencing growth and muscle development [40].

In turn, in CMS, a ROH island encompassing the *ASIP* gene, a determinant of coat color in mammals, was identified. This can potentially highlight the importance of selection for pigmentation traits. Research by Zhang et al. [38] and Koseniuk et al. [41] has shown significant variation within the *ASIP* locus between white and black sheep, with strong selection signals observed for white versus non-white coat pigmentation [42]. This aligns with the selection criteria for CMS, introduced in 1999, where coat color has been a key trait under genetic resource protection in Poland [43]. The observed genome signals at the *ASIP* locus confirm the breed’s adherence to its original selection goals, ensuring the preservation of its unique genetic pool.

A gene of potential interest is *EEF1AKMT1*, identified within ROH hotspots on chromosome 10 in PMS, PZ, and CMS breeds, which shared an extensive ROH island. *EEF1AKMT1* is responsible for modifying elongation factors, which are critical in protein synthesis processes that potentially influence growth and reproductive traits across various species, including sheep [44]. Although direct evidence connecting *EEF1AKMT1* with specific performance traits in sheep remains limited, its involvement in genetic interactions and metabolic pathways may indirectly affect important production traits. Previous research highlights the significance of genes associated with metabolic functions in livestock productivity. For instance, Li et al. [42] identified genetic markers related to wool efficiency in sheep and proposed that genes involved in protein synthesis might significantly influence key production traits, indirectly supporting the relevance of *EEF1AKMT1*.

In conclusion, this study provides insights into the genetic architecture of Polish-maintained sheep breeds, revealing significant variation in the patterns of runs of homozygosity and heterozygosity. The analysis of ROH and ROHet segments across breeds highlighted the influence of historical selection pressures and management strategies on genomic diversity. Breeds such as SW and MPC exhibited high mean sums of ROH, especially formed due to the presence of long ROH segments, potentially reflecting recent inbreeding. Conversely, breeds like PZ and PMS maintained greater genetic diversity, likely due to a broader genetic background. The presence of ROH islands associated with key production and reproductive traits—including milk yield, body condition, and lambing potential—underscores the functional importance of these genomic regions. Genes located within these ROH hotspots, such as *SPP1*, *ABCG2*, and members of the small nuclear RNA family, highlight the complex interplay between the selection for economic traits and the conservation of genetic variation. Moreover, the results align with global studies on sheep populations, illustrating common patterns of selection, such as the prevalence of short ROH segments from historical inbreeding and the accumulation of long ROH in intensively managed breeds.

## Figures and Tables

**Table 1:** Detailed information about the sheep breeds maintained in Poland.

Breed	Breed Abbreviation	Number of Individuals	Type of Use	Wool Type	Reproduction
Polish Mountain Sheep	PMS	104	Multi-purpose	Mixed	Low
Podhale Zackel	PZ	100	Multi-purpose	Mixed	Low
Colored Mountain Sheep	CMS	97	Multi-purpose	Mixed	Low
Swiniarka	SW	99	Multi-purpose	Mixed	Low
Uhruska	UHR	69	Wool and meat	Solid	Medium
Old-type Merino	MPOT	50	Wool and meat	Solid	Medium
Polish Merino of Colored Variety	MPC	59	Wool and meat	Solid	Medium
Black-headed	BH	104	Meat	Solid	Medium
Wrzosówka	WRZ	90	Sheepskin	Mixed	High

Table 1 was created according to Gurgul et al. [15]. “Reproduction” levels reflect average litter size as an indicator of prolificacy, classified according to Gurgul et al. [15]. “Low” indicates ≤1.5 lambs per lambing, “Medium” indicates 1.6–1.9, and “High” indicates ≥2 lambs per lambing.

## Data Availability

No new data were created or analyzed in this study. Data sharing is not applicable to this article.

## Supplementary Materials

The following supporting information can be downloaded at: https://www.mdpi.com/article/10.3390/genes16060709/s1, Supplementary Material S1. Detailed statistics regarding ROH and ROHet, FROH and DROHet; Supplementary Material S2. The numbers and lengths of identifed ROH calculated for each breed and each individual; Supplementary Material S3. The identified ROH islands with annotation of genes. Supplementary Material S4. The overlap of ROH islands with a QTL database.
