# Supplementary material for: Exploring Runs of Homozygosity and Heterozygosity in Sheep Breeds Maintained in Poland
Source: Genes (Basel). 2025 Jun 14;16(6):709. doi: 10.3390/genes16060709 (PMC12192788; doi:10.3390/genes16060709)
Supplement: Supplementary file 1 [file genes-16-00709-s001.zip › Supplementary Material S1.pdf]

Table S1. Summary statistics of ROH across different length categories in various sheep breeds

		Stat	ROH length category (Mb)				
			>1	>2	>4	>8	>16
PMS	Number/animal	**Mean**	**24.29**	**17.68**	**8.94**	**2.86**	**0.45**
		SD	12.06	10.05	6.63	3.37	1.29
		Min	7	2	1	0	0
		Max	61	54	44	26	11
	Length/animal (Mb)	**Mean**	**104.16**	**93.57**	**69.14**	**35.24**	**9.65**
		SD	75.23	72.87	65.27	48.98	28.65
		Min	17.33	4.28	5.58	0	0
		Max	575.44	561.84	533.33	423.30	255.71
PZ	Number/animal	**Mean**	**13.11**	**8.42**	**3.44**	**0.88**	**0.13**
		SD	5.21	4.14	3.15	1.57	0.66
		Min	5	2	0	0	0
		Max	37	29	23	10	6
	Length/animal (Mb)	**Mean**	**48.82**	**39.40**	**25.68**	**11.67**	**3.43**
		SD	40.20	39.15	37.92	29.75	21.19
		Min	10.98	5.04	0	0	0
		Max	360.07	347.41	331.29	255.36	204.69
CMS	Number/animal	**Mean**	**18.22**	**13.22**	**7.13**	**3.56**	**1.37**
		SD	8.68	7.51	5.91	4.20	2.36
		Min	1	1	0	0	0
		Max	41	35	25	20	15
	Length/animal (Mb)	**Mean**	**103.55**	**96.30**	**79.12**	**59.02**	**34.50**
		SD	93.53	92.54	89.30	81.21	64.11
		Min	2.41	2.41	0	0	0
		Max	566.20	559.4	542.88	510.28	455.76
SW	Number/animal	**Mean**	**52.49**	**45.72**	**32.57**	**18.20**	**6.88**
		SD	11.97	11.76	9.91	7.55	4.93
		Min	17	15	9	4	1
		Max	82	75	63	47	25
	Length/animal (Mb)	**Mean**	**433.42**	**422.38**	**384.00**	**302.28**	**175.63**
		SD	184.32	185.12	183.32	175.21	153.18
		Min	100.83	97.88	85.16	44.46	17.03
		Max	1152.14	1146.05	1101.29	1037.86	854.75
UHR	Number/animal	**Mean**	**45.01**	**31.29**	**14.23**	**5.18**	**0.78**
		SD	10.13	9.09	6.48	3.38	1.11
		Min	25	14	1	0	0
		Max	64	48	30	16	6
	Length/animal (Mb)	**Mean**	**184.56**	**162.32**	**114.35**	**64.61**	**16.75**
		SD	68.26	67.98	62.85	47.40	24.26
		Min	68.11	45.49	6.96	0	0
		Max	382.74	360.31	311.71	232.44	126.31
MPOT	Number/animal	**Mean**	**57.68**	**36.20**	**11.16**	**2.38**	**0.36**
		SD	8.52	7.62	3.75	1.73	0.63
		Min	40	19	4	0	0
		Max	77	52	19	6	2
	Length/animal (Mb)	**Mean**	**179.59**	**145.11**	**76.20**	**29.01**	**8.13**
		SD	38.32	38.39	31.26	25.18	15.83
		Min	111.08	72.42	26.66	0	0
		Max	266.40	228.22	147.79	100.99	72.31
MPC	Number/animal	**Mean**	**86.98**	**63.20**	**29.55**	**11.61**	**3.06**
		SD	9.95	10.63	8.20	6.47	3.99
		Min	63	40	18	4	0
		Max	111	92	64	42	23
	Length/animal (Mb)	**Mean**	**398.23**	**359.57**	**265.60**	**166.52**	**73.05**
		SD	137.88	141.44	141.32	134.71	110.27

		Min	232.33	194.14	122.19	42.88	0
		Max	1045.29	1024.36	947.43	839.45	607.95
**BH**	Number/animal	**Mean**	**44.42**	**28.53**	**11.09**	**3.73**	**1.21**
		SD	7.74	6.53	5.10	3.81	1.93
		Min	27	14	2	0	0
		Max	69	59	44	32	16
	Length/animal (Mb)	**Mean**	**172.07**	**146.63**	**98.01**	**58.13**	**29.59**
		SD	79.07	79.95	78.57	74.05	54.49
		Min	82.15	56.12	10.55	0	0
		Max	760.24	751.34	708.65	638.11	452.18
**WRZ**	Number/animal	**Mean**	**43.41**	**36.67**	**22.25**	**9.14**	**2.13**
		SD	9.98	8.85	6.42	3.71	1.73
		Min	22	20	10	1	0
		Max	89	72	48	19	6
	Length/animal (Mb)	**Mean**	**249.20**	**238.35**	**196.46**	**122.50**	**45.23**
		SD	67.93	66.94	62.82	53.21	37.86
		Min	131.63	124.36	89.44	11.70	0
		Max	469.02	441.01	369.72	282.73	148.11

PMS-Polish Mountain Sheep; PZ- Podhale Zackel; CMS- Colored Mountain Sheep; SW- Swiniarka; UHR- Uhruska; MPOT- Old-type Merino; MPC- Polish Merino of Colored Variety; BH- Black-headed; WRZ- Wrzosówka

Table S2. The proportion of the genome covered by runs of homozygosity (Table A - F_{ROH}) and runs of heterozygosity (Table B - D_{ROHet}) across different ROH length categories and sheep breeds.

Table A.

**Breed**	**FROH >1 Mb**	**FROH >2 Mb**	**FROH >4 Mb**	**FROH >8 Mb**	**FROH >16 Mb**
PMS	0.04	0.03599	0.02659	0.01355	0.00371
PZ	0.018	0.015155	0.009876	0.004491	0.001322
CMS	0.039831	0.037039	0.030433	0.0227	0.01327
SW	0.166704	0.162454	0.147692	0.116261	0.067551
UHR	0.070987	0.062433	0.043981	0.02485	0.006445
MPOT	0.04	0.03599	0.02659	0.01355	0.00371
MPC	0.018	0.015155	0.009876	0.004491	0.001322
BH	0.039831	0.037039	0.030433	0.0227	0.01327
WRZ	0.166704	0.162454	0.147692	0.116261	0.067551

Table B.

**Breed**	**D_{ROHet} >0.5 Mb**	**D_{ROHet} >1 Mb**	**D_{ROHet} >1.5 Mb**
PMS	0.003235	0.002626	0.000967
PZ	0.002963	0.002475	0.000777
CMS	0.003024	0.000768	0.000298
SW	0.002817	0.002304	0.000525
UHR	0.003908	0.00359	0.001677
MPOT	0.002794	0.002187	0.000348
MPC	0.002679	0.001703	0.000429
BH	0.003623	0.000732	0.00019
WRZ	0.002924	0.002247	0.000744

PMS-Polish Mountain Sheep; PZ- Podhale Zackel; CMS- Colored Mountain Sheep; SW- Swiniarka; UHR- Uhruska; MPOT- Old-type Merino; MPC- Polish Merino of Colored Variety; BH- Black-headed; WRZ- Wrzosówka

Table S3. Summary statistics of ROHet across different length categories in the studied sheep breeds

		**Stat**	**ROHet length category (Mb)**		
			**>0.5 Mb**	**>1 Mb**	**>1.5 Mb**
**PMS**	Number/animal	**Mean**	10.53	8.34	2.85
		SD	3.39	5.81	5.35
		Min	4	0	0
		Max	20	20	20
	Length/animal (Mb)	**Mean**	8.41	6.82	2.51
		SD	2.98	4.80	4.69
		Min	3.11	0	0
		Max	17.03	17.03	17.03
**PZ**	Number/animal	**Mean**	9.55	7.75	2.17
		SD	2.79	4.91	4.35
		Min	4	0	0
		Max	15	15	15
	Length/animal (Mb)	**Mean**	7.70	6.43	2.02
		SD	2.43	4.06	4.05
		Min	3.28	0	0
		Max	15.01	15.01	15.01
**CMS**	Number/animal	**Mean**	9.75	1.49	0.45
		SD	3.36	1.18	0.72
		Min	2	0	0
		Max	18	6	4
	Length/animal (Mb)	**Mean**	7.86	1.99	0.77
		SD	2.89	1.65	1.23
		Min	1.21	0	0
		Max	15.42	7.98	6.82
**SW**	Number/animal	**Mean**	9.04	7.17	1.54
		SD	2.97	4.84	3.76
		Min	3	0	0
		Max	19	19	14
	Length/animal (Mb)	**Mean**	7.32	5.99	1.36
		SD	2.55	4.03	3.31
		Min	2.53	0	0
		Max	14.35	14.35	11.80
**UHR**	Number/animal	**Mean**	12.81	11.65	5
		SD	3.79	5.69	7.22
		Min	2	0	0
		Max	22	22	22
	Length/animal (Mb)	**Mean**	10.16	9.33	4.36
		SD	3.36	4.71	6.29
		Min	1.59	0	0
		Max	19.44	19.44	19.44
**MPOT**	Number/animal	**Mean**	9.38	7.12	1.06
		SD	2.70	5.01	3.03
		Min	5	0	0
		Max	16	16	12
	Length/animal (Mb)	**Mean**	7.26	5.68	0.90
		SD	2.17	3.97	2.56
		Min	3.38	0	0
		Max	12.46	12.46	9.54

**MPC**	Number/animal	**Mean**	9.08	5.55	1.30
		SD	3.25	5.50	3.49
		Min	2	0	0
		Max	15	15	15
	Length/animal (Mb)	**Mean**	6.96	4.42	1.11
		SD	2.57	4.39	2.97
		Min	1.12	0	0
		Max	12.65	12.65	12.65
**BH**	Number/animal	**Mean**	11.96	1.40	0.25
		SD	3.15	1.12	0.50
		Min	5	0	0
		Max	20	5	2
	Length/animal (Mb)	**Mean**	9.41	1.90	0.49
		SD	2.50	1.60	0.98
		Min	4.01	0	0
		Max	15.99	7.02	4.74
**WRZ**	Number/animal	**Mean**	9.71	7.25	2.28
		SD	3.58	5.85	4.68
		Min	3	0	0
		Max	22	22	16
	Length/animal (Mb)	**Mean**	7.60	5.84	1.93
		SD	2.83	4.62	3.91
		Min	2.22	0	0
		Max	17.00	17.00	12.05

PMS-Polish Mountain Sheep; PZ- Podhale Zackel; CMS- Colored Mountain Sheep; SW- Swiniarka; UHR- Uhruska; MPOT- Old-type Merino; MPC- Polish Merino of Colored Variety; BH- Black-headed; WRZ- Wrzosówka
