# Supplementary material for: Exploring Runs of Homozygosity and Heterozygosity in Sheep Breeds Maintained in Poland
Source: Genes (Basel). 2025 Jun 14;16(6):709. doi: 10.3390/genes16060709 (PMC12192788; doi:10.3390/genes16060709)
Supplement: Supplementary file 1 [file genes-16-00709-s001.zip › Supplementary Material S4.pdf]

Breed	Position			Size	QTL type
	Chr	Start	Stop		
PMS	10	43959538	44959973	1000436	Semen volume QTL (283340)
	19	46882636	47063567	180932	Lambing potential QTL (263657)
	2	112766526	115525483	2758958	Body condition score QTL (263630)
	2	115887593	116096980	209388	Body condition score QTL (263630)
PZ	10	41420084	45133795	3713712	Semen volume QTL (283340)
CMS	1	111892027	113034032	1142006	Clean fleece weight QTL (263609)
	1	273726534	274195436	468903	Muscle density QTL (95880)
	10	40749405	45677506	4928102	Semen volume QTL (283340)
	13	62721325	64718393	1997069	Muscle pH QTL (282955)
	15	8757097	12253686	3496590	Muscle pH QTL (282971)
	19	45929701	46056473	126773	Lambing potential QTL (263657)
	2	114411448	115912934	1501487	Body condition score QTL (263630)
	9	31421304	33354903	1933600	Mean fiber diameter QTL (263605)
SW	1	184125364	184855004	729641	Body condition score QTL (263629)
	1	185028417	186094491	1066075	Body condition score QTL (263629)
	10	43417893	44708676	1290784	Semen volume QTL (283340)
	15	4679104	8541933	3862830	Muscle pH QTL (282970)
	22	38526561	38836171	309611	Carcass bone percentage QTL (95878)<br>Muscle density QTL (95879)
	8	76392721	76414104	21384	Sperm motility QTL (283364)
	9	44665941	45711860	1045920	Mean fiber diameter QTL (263605)
	9	78607295	80649238	2041944	Sperm motility QTL (283367)
	9	80781440	82992075	2210636	Clean fleece weight QTL (263616)
UHR	2	113422825	114397648	974824	Body condition score QTL (263630)
	2	114676373	114861327	184955	Body condition score QTL (263630)
MPOT	1	236746611	237637304	890694	Mean fiber diameter QTL (263599)
MPC	1	227831862	227903221	71360	Mean fiber diameter QTL (263599)
	11	53352516	56094938	2742423	Curd firming time QTL (224816)
	19	19166441	22588766	3422326	Lambing potential QTL (263657)
	8	43386232	44935866	1549635	Mean fiber diameter QTL (263603)
	8	45066181	46382226	1316046	Mean fiber diameter QTL (263603)
BH	1	227525521	227692672	167152	Mean fiber diameter QTL (263599)
	1	228318069	228425113	107045	Mean fiber diameter QTL (263599)
	25	7599609	7694641	95033	Semen volume QTL (283344)
	9	28120707	28233297	112591	Rennet coagulation time QTL (224815)
WRZ	1	227411968	228985580	1573613	Mean fiber diameter QTL (263599)
	2	113449517	114763217	1313701	Body condition score QTL (263630)
	25	13063841	13795161	731321	Sperm motility QTL (283371)
